# Supplementary material for: Experimental Demonstration of Anomalous Field Enhancement in All-Dielectric Transition Magnetic Metamaterials
Source: Sci Rep. 2015 Nov 4;5:16154. doi: 10.1038/srep16154 (PMC4632012; doi:10.1038/srep16154)
Supplement: Supplementary Information [file srep16154-s1.pdf]

**Supplementary Materials:**

# Experimental Demonstration of Anomalous Field Enhancement in All-Dielectric Transition Magnetic Metamaterials

Jingbo Sun[1], Xiaoming Liu[2], Ji Zhou[2]*, Zhaxylyk Kudyshev[1], and Natalia M. Litchinitser[1]*

1 Department of Electrical Engineering, University at Buffalo The State University of New York, Buffalo, NY 14260, USA.

2 State Key Laboratory of New Ceramics and Fine Processing, School of Materials Science and Engineering, Tsinghua University, Beijing 100084, People's Republic of China

Correspondence and requests for materials should be addressed to natashal@buffalo.edu and zhouji@mail.tsinghua.edu.cn

**The loss effect inside the transition metamaterial:** In our experimental studies, we measured the power distribution in the *xy*-plane, as shown in Supplementary Fig. 1(a). The magnetic field components, $H_x$ which is the component along the normal to the zero$-\mu$ interface and $H_y$ which is the component along the zero-$\mu$ interface of the metamaterials, can be calculated, using Maxwell's equations, as

$$H_x = -\frac{ic}{\omega\mu(x)}\frac{\partial E_z}{\partial y},\ H_y = \frac{ic}{\omega\mu(x)}\frac{\partial E_z}{\partial x}. \qquad \text{(S1)}$$

For the case of constant $\varepsilon$ and linearly changing $\mu$, following[1], we can find $H_x \sim \frac{1}{x}$ and $H_y \sim \ln \alpha x$, where $\alpha = \sin^2(\theta)$. The distributions of $H_x$ and *Hy,* calculated from measured $E_z$, are shown in Supplementary Fig. 1(b) and (c). Using Comsol Multiphysics 4.3b, we also performed the simulation on such a transition metamaterial with effective parameters shown in Fig. 2(b). Numerical results, shown in Supplementary Fig. 1(d-f), agree well with the experimental results.

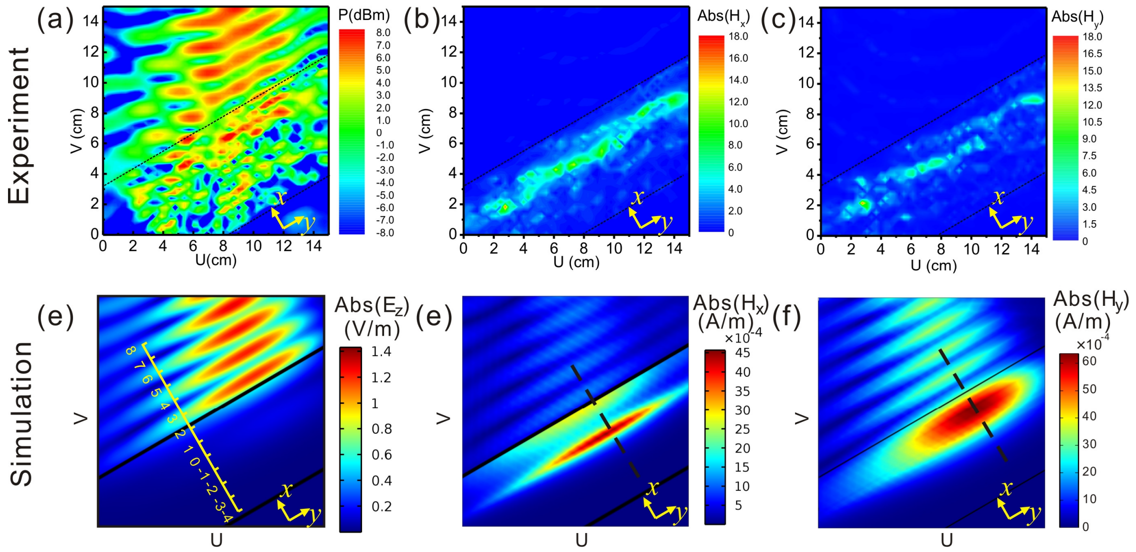

Supplementary Figure 1. Experimental results: (a) power, (b) amplitude of $H_x$, (c) amplitude of $H_y$. Simulation results, based on the parameters from the retrieval method: (d) amplitude of $E_z$, (e) amplitude of $H_x$, (f) amplitude of $H_y$. The scale in (d) shows the size of the transition metamaterial along $x$ axis (in cm), corresponding to that used in the experiment.

Figure 2 in the main text shows the real part of magnetic permeability retrieved for our metamaterials sample. Supplementary Fig. 2 shows the imaginary part of magnetic permeability. In particular, the imaginary part of the permeability is equal to 0.021 at the point where the real part is equal to zero (corresponding to the unit cell size c=5.6mm). Despite the theoretical prediction of a stronger enhancement for the $H_x$ component as compared to that for $H_y$, Supplementary Fig. 1(e) and (f) show comparable maximum values of $H_x$ and $H_y$.

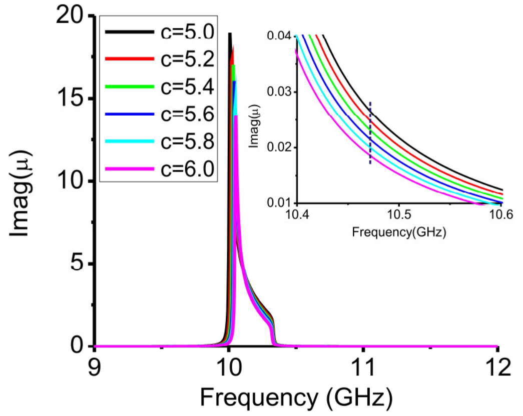


Supplementary Figure 2. Imaginary part of the transition metamaterial from the retrieval method.

In order to explain this result, we studied the effect of material losses of our transition metamaterial on these two components of the magnetic field. We performed numerical studies of the transition material with the same real part of magnetic permeability and its profile, but different imaginary parts, ranging from 0.0035 to 0.033 at the zero-$\mu$ point. Supplementary

Figure 3 shows the cross-sections of the $H$-field distribution along the dash lines in Supplementary Fig. 1(e) and (f) for different imaginary parts of $\mu$. As shown in Supplementary Fig. 3, for the low-loss case, the peak value of $H_x$ is significantly larger than $H_y$ around the zero permeability region, in agreement with the theoretical predictions. As the losses increase, $H_x$ is decreasing and may become even smaller than $H_y$. Although the absolute value of the imaginary part of $\mu$ is not relatively low in its absolute value as compared to its real part, far from $\mu$=0, $x$=0, at the point $x$=0, the real part of $\mu$ is zero, and therefore, the contribution of the imaginary part of $\mu$ becomes significant. The simulation results shown in Supplementary Fig. 1(e) and (f) correspond to the yellow lines in Supplementary Fig. 3, explaining the fact that normal and tangential components are comparable. However, the experimental results in Supplementary Fig. 1(b) and (c) show strongly enhanced $H_x$ that is larger than $H_y$, suggesting that the actual losses in our sample are lower than those obtained from the parameter retrieval procedure.

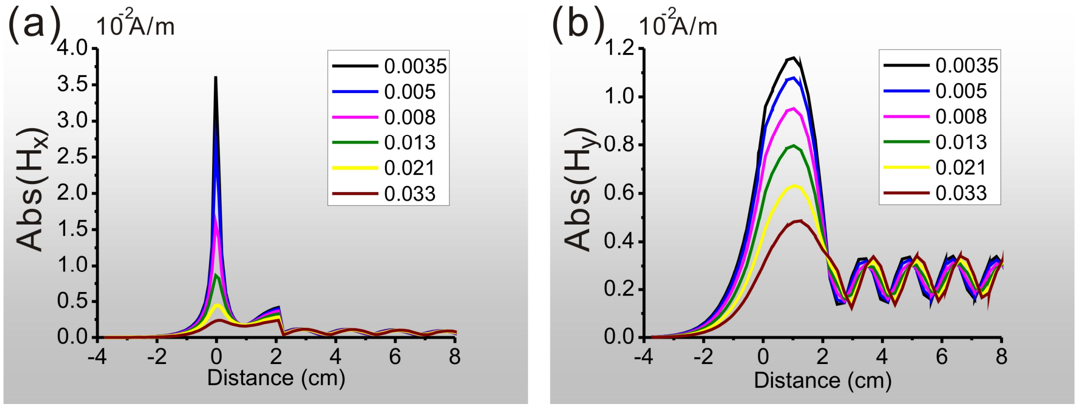


Supplementary Figure 3. Magnetic field distribution along the direction normal to zero-$\mu$ interface, with different loss: (a) $H_x$ and (b) $H_y$. The data is taken along the dash line marked in Supplementary Fig. 1(e) for $H_x$ and Supplementary Fig. 1(f) for $H_y$. The zero permeability point is estimated to be around 2.4cm away from the ($\mu$=0, $x$=0), which corresponds to the c=5.6 mm cube array.

**Normal incidence:** Finally, we performed the measurements for the case of normal incidence of the beam ($\theta$=0). As expected, there is a total reflection at the zero permeability interface (as $n$=0 when $\mu$=0). The upper boundary of the sample is at V=7.5cm. As shown in Supplementary Fig. 4(c), the beam is reflected around V=5.4cm, which further confirms the spatial location of the zero permeability position. In the case of normal incidence of the Gaussian beam along the $x$ direction, with E field along the $z$ direction, the H field is mainly pointing along the $y$ direction. Therefore, at least in the center of the Gaussian beam, there is no longitudinal component of magnetic field[2]. However, due to the finite wave-vector distribution in the Gaussian beam, there will be some $H_x$ components on the periphery of the beam, where the wave vectors are not exactly along the $x$ direction. Therefore, the $H_x$ field will be enhanced in those regions, similar to the oblique incidence case, as indicated in Supplementary Fig. 4(b).

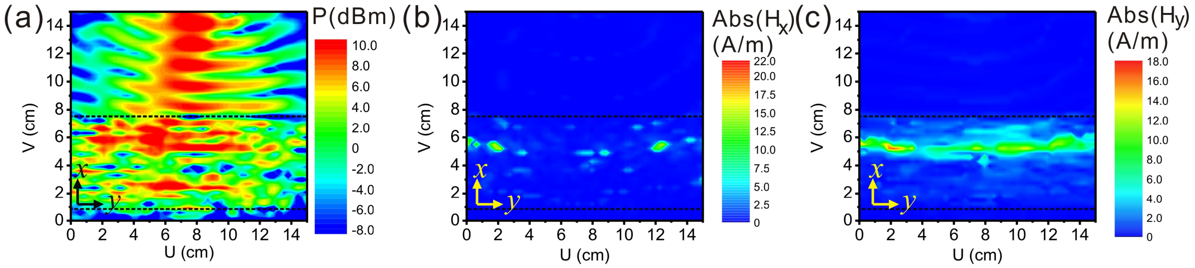


Supplementary Figure 4. Normal incidence case: (a) power distribution; (b) amplitude of $H_x$; (c) amplitude of $H_y$.

## Supplementary References


[1] Landau, L.D., Pitaevski, L. P., Lifshitz, E.M. *Electrodynamics of Continuous Media, Second Edition: Volume 8 (Course of Theoretical Physics S) 2 edition*, (Robert Maxwell, M.C, 1984).

[2] Alali, F. and Litchinitser, N. M. Gaussian beams in near-zero transition metamaterials. *Opt. Commun.* **291**, 179-183 (2013).
